# Benzaldehyde Thiosemicarbazone Derived from Limonene Complexed with Copper Induced Mitochondrial Dysfunction in *Leishmania amazonensis*

**DOI:** 10.1371/journal.pone.0041440

**Published:** 2012-08-01

**Authors:** Elizandra Aparecida Britta, Ana Paula Barbosa Silva, Tânia Ueda-Nakamura, Benedito Prado Dias-Filho, Cleuza Conceição Silva, Rosana Lázara Sernaglia, Celso Vataru Nakamura

**Affiliations:** 1 Programa de Pós-graduação em Ciências Farmacêuticas, Universidade Estadual de Maringá, Paraná, Brazil; 2 Departamento de Química, Universidade Estadual de Maringá, Maringá, Paraná, Brazil; Albert Einstein College of Medicine, United States of America

## Abstract

**Background:**

Leishmaniasis is a major health problem that affects more than 12 million people. Treatment presents several problems, including high toxicity and many adverse effects, leading to the discontinuation of treatment and emergence of resistant strains.

**Methodology/Principal Findings:**

We evaluated the *in vitro* antileishmanial activity of benzaldehyde thiosemicarbazone derived from limonene complexed with copper, termed BenzCo, against *Leishmania amazonensis*. BenzCo inhibited the growth of the promastigote and axenic amastigote forms, with IC_50_ concentrations of 3.8 and 9.5 µM, respectively, with 72 h of incubation. Intracellular amastigotes were inhibited by the compound, with an IC_50_ of 10.7 µM. BenzCo altered the shape, size, and ultrastructure of the parasites. Mitochondrial membrane depolarization was observed in protozoa treated with BenzCo but caused no alterations in the plasma membrane. Additionally, BenzCo induced lipoperoxidation and the production of mitochondrial superoxide anion radicals in promastigotes and axenic amastigotes of *Leishmania amazonensis*.

**Conclusion/Significance:**

Our studies indicated that the antileishmania activity of BenzCo might be associated with mitochondrial dysfunction and oxidative damage, leading to parasite death.

## Introduction

Leishmaniasis is still considered a major health problem, with high morbidity and mortality and affecting more than 12 million people. The size of the population at risk is approximately 350 million [1]. Leishmaniasis transmission occurs through hematophagous vectors of the genera *Lutzomia* and *Phlebotomus* in the New and Old Worlds, respectively. The life cycle of *Leishmania* species includes an intracellular amastigote within the mononuclear phagocytes in vertebrate hosts and an extracellular promastigote form in insect vectors [2], [3]. No vaccines are effective against these diseases, and treatment depends on a limited range of drugs [4].

Thiosemicarbazones and their metallic complexes are an important class of compounds that have been extensively studied in recent years, mainly because of their broad profile of pharmacological activity [5]. Several studies have demonstrated the chemotherapeutic properties of these compounds, including antitumor, antibacterial, antiviral, and antiprotozoal activity [6]–[10]. Generally, the mechanisms of action of these compounds involve inhibition of the enzyme by forming endogenous metal complexes or a redox reaction, DNA interactions, or DNA synthesis inhibition [11]. Moreover, thiosemicarbazones or metallic complexes mimic the action of enzymes as a copper complex (II), reproducing the superoxide dismutase [12].

In the present study, we evaluated the antileishmanial activity of the benzaldehyde thiosemicarbazone derived from limonene complexed with copper, termed BenzCo, against the promastigote and axenic amastigote forms of *L. amazonensis,* its effects on the interaction of this flagellate with mouse peritoneal macrophages, and its intracellular effects that could lead to parasite death.

**Figure 1:**
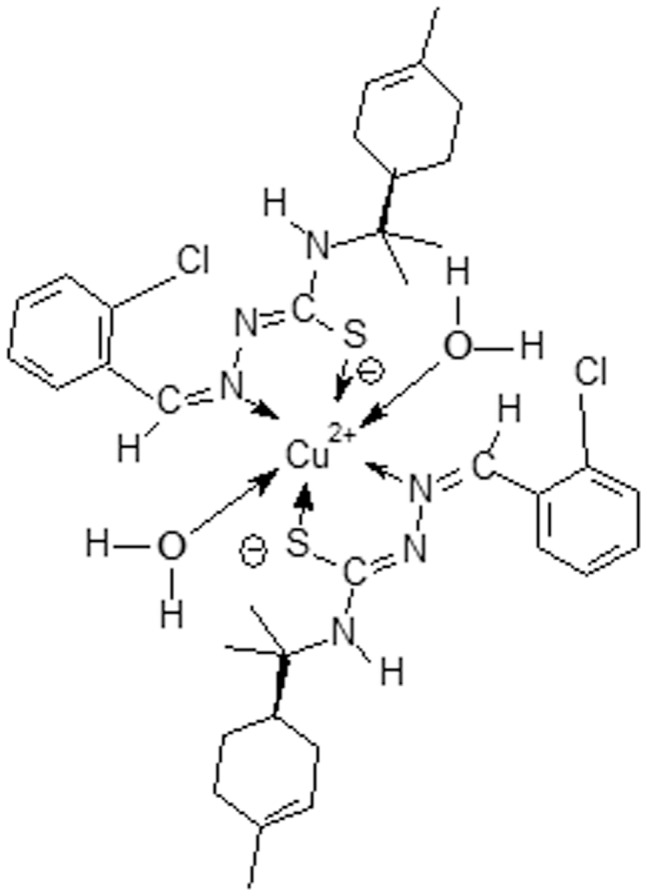
Proposed structure of the [(diaquo) bis {[*N*(4)-(*R*-(1-methyl-4-isopropyl)-cyclohexene]-2-*o*-chlorobenzaldehyde thiosemicarbazonate} copper (II)] complex.

## Materials and Methods

### 1. Chemistry

All melting points were determined using a Microquímica model MQAPF-301 apparatus. Conductance values were obtained in a Fenton mCA 150 at 298 K from 10^−3^ mol L^−1^ in absolute EtOH. Electronic spectra were recorded with a spectrophotometer Varian, Cary-50 in CHCl_3_ solution. Infrared spectra were obtained using KBr pellets in an FT-IR BOMEM spectrophotometer. Low-resolution mass spectra were recorded by means of a SHIMADZU-CG/MS model QP 2000A spectrometer at 70 eV with a probe for solids. The optical rotations were determined in CHCl_3_ as a solvent with a Perkin-Elmer polarimeter 343 model at 25°C. Proton nuclear magnetic resonance (^1^H NMR) spectra were recorded using CDCl_3_ as a solvent at ambient temperature using a Varian Mercury spectrometer (300 MHz) with TMS as an internal standard.

**Figure 2:**
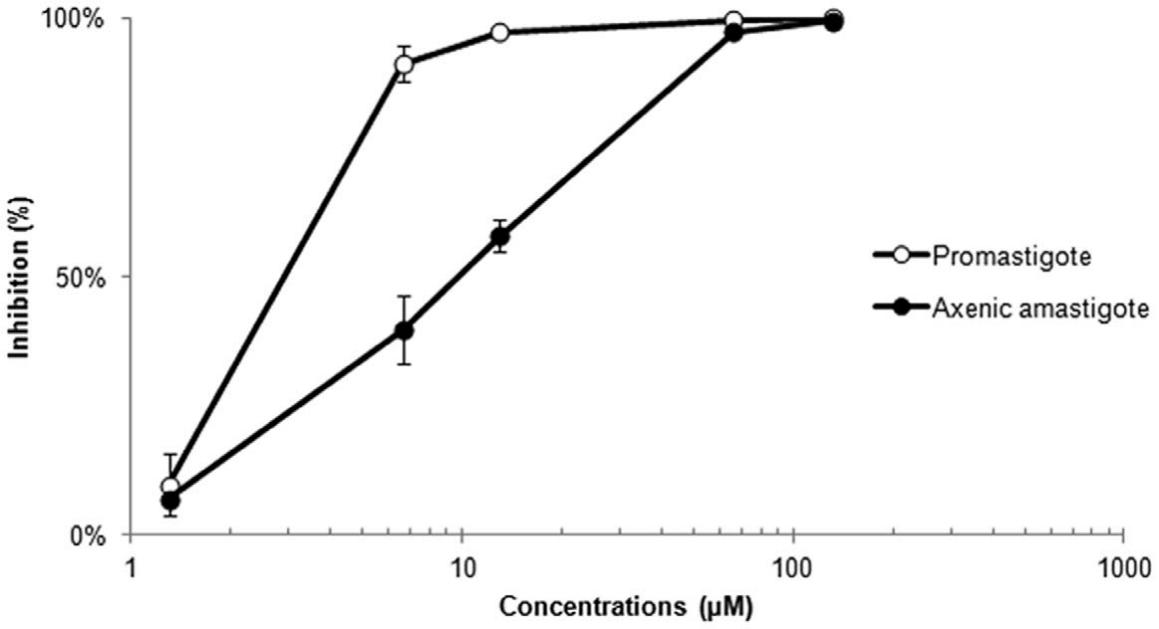
Inhibition percentage of promastigote and axenic amastigote forms of *Leishmania amazonensis* treated with BenzCo for 72 h in culture. The data are expressed as the means from three independent tests. All of the results were statistically significant at *p*<0.05 when compared with the control group (Kruskal-Wallis test).

### 2. Procedure for Synthesis of Benzaldehyde Thiosemicarbazone

For the synthesis of *N*(4)-[R-(1-methyl-4-isopropyl)-cyclohexene]-2-*o*-chlorobenzaldehyde thiosemicarbazone, 2.86 mmol (0.65 g) of *N*(4)-[R-(1-methyl-4-isopropyl-cyclohexene]-thiosemicarbazide [13] dissolved in CHCl_3_ was added to a solution of *o*-chlorobenzaldehyde (2.86 mmol) in CHCl_3_ and drops of trifluoracetic acid. After 30 min at room temperature with stirring the solid product was filtered and recrystallized from ethanol (white crystals; yield, 90%; melting point, 161–164°C; [α]_D_ +33). UV (CHCl_3_ solution), λ_max/nm_: 323+sh. IR (KBr): (NH) 3322 and 3149, (C = N) 1595, (C = S) 804; EI-MS *m/z* 349 (M+•). ^1^H NMR (300 MHz, CDCl_3_): δ 10.07 (1H, s, NH, H-2), 7.54 (1H, s, NH, H-4), 5.39 (1H, brs, H-2′), 1.80–2.05 (2H, m, H-3′), 2.73 (1H, m, H-4′), 1.85 (2H, m, H-5′), 2.01–2.11 (2H, m, H-6′), 1.54 (3H, s, H-8′), 1.51 (3H, s, H-9′), 1.66 (3H, s, H-10′), 8.27 (1H, s, HC = N, H-1a), 7.38 (1H, m, H-3a), 7.30 (2H, m, H-4a/H-5a), 7.80 (1H, m, H-6). ^13^C NMR (75.5 MHz, CDCl_3_): δ 134.3 (C-1′), 120.6 (C-2′), 26.8 (C-3′), 41.0 (C-4′), 24.4 (C-5′), 31.3 (C-6′), 59.1 (C-7′), 24.5 (C-8′), 24.2 (C-′), 23.5 (C-10′), 138.0 (C = N, C-1′′), 131.1 (C-2′′), 130.4 (C-3′′), 127.2 (C-4′′), 131.2 (C-5′′), 127.0 (C-6′′), 134.6 (C-7′′), and 174.8 (C = S, C-3) [14].

**Figure 3:**
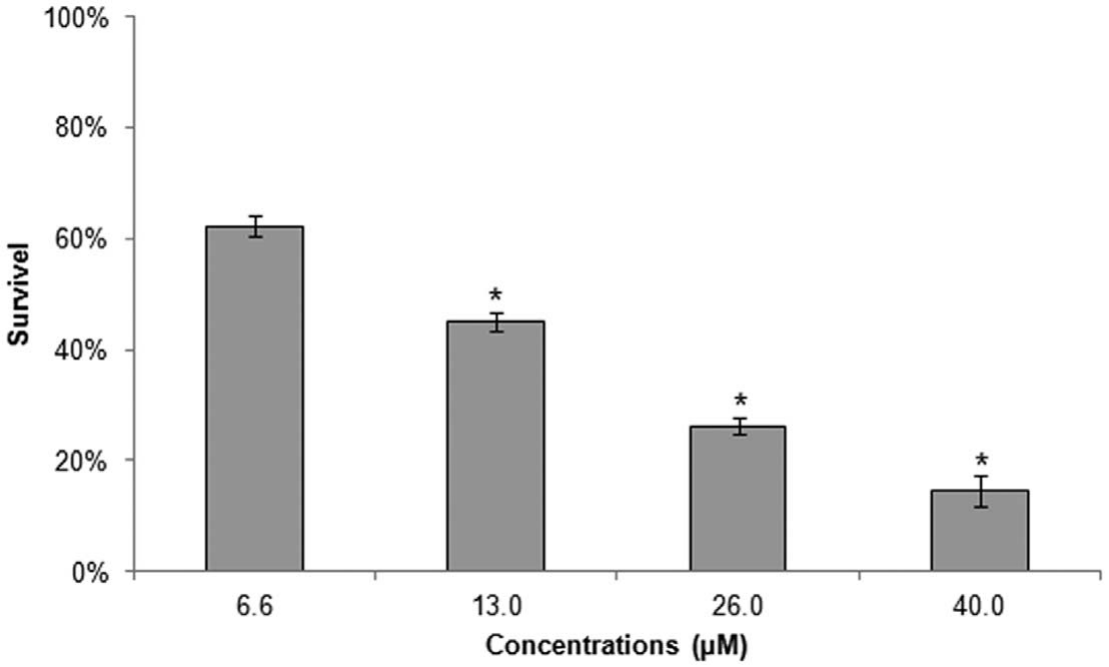
Effect of BenzCo on the interaction between *Leishmania amazonensis* and mouse peritoneal macrophages. The survival percentage was calculated by multiplying the percentage of infected macrophages by the mean number of internalized parasites per infected macrophage. The data are expressed as the means from three independent experiments. *p<0.05, compared with the control group.

### 3. Procedure for Synthesis of Benzaldehyde Thiosemicarbazone Complexed with Copper

For the synthesis of the [(diaquo) bis {[*N*(4)-(*R*-(1-methyl-4-isopropyl)-cyclohexene]-2-*o*-chlorobenzaldehyde thiosemicarbazonate} copper (II)] (BenzCo), solid CuCl_2_ (0.075 mmol, 13 mg) was added to a solution of ligand (*N*(4)-[R-(1-methyl-4-isopropyl)-cyclohexene]-2-*o*-chlorobenzaldehyde thiosemicarbazone) (0.15 mmol, 54.75 mg) in 20 ml of 95% MeOH, at room temperature with stirring, in a 1∶2 Cu:ligand molar ratio. After 30 min, a precipitate appeared. The mixture was filtered and the precipitate was successively washed with absolute ethanol. The alcoholic solution was filtered to remove the insoluble part and the slow evaporation of the filtrate gave a solid. It was washed with CHCl_3_ and dried in vacuum over silicagel, furnishing copper complex as a green powder with a 73% yield. FW: 827.5 gmol^−1^. UV (CHCl_3_ solution), λ_max/nm_: 316, 518, and 842. IR cm^−1^: (KBr) ν_max/cm_^−1^: (H_2_0) 3437, (NH) 3204, (H_2_0) 1625, (C = N) + (C = C) 1565, 1537, (CS) 1358, 731, (Cu-N) 462. Molar conductivity (1×10^−3^ mol L^−1^) ethanol: 5.4 Ω^−1^ cm^2^ mol^−1^ (Fig. 1).

### 4. Parasites

*Leishmania amazonensis* promastigotes (MHOM/BR/Josefa) were maintained at 25°C in Warren’s medium (brain-heart infusion plus hemin and folic acid; pH 7.2) supplemented with 10% heat-inactivated fetal bovine serum (FBS; Gibco Invitrogen, Grand Island, NY, USA). Axenic amastigotes were obtained by the *in vitro* transformation of infective promastigotes by a progressive temperature increase and pH decrease [15]. These forms were maintained in Schneider’s medium (Sigma, St. Louis, MO, USA), pH 4.6, that contained 20% FBS at 32°C.

**Figure 4:**
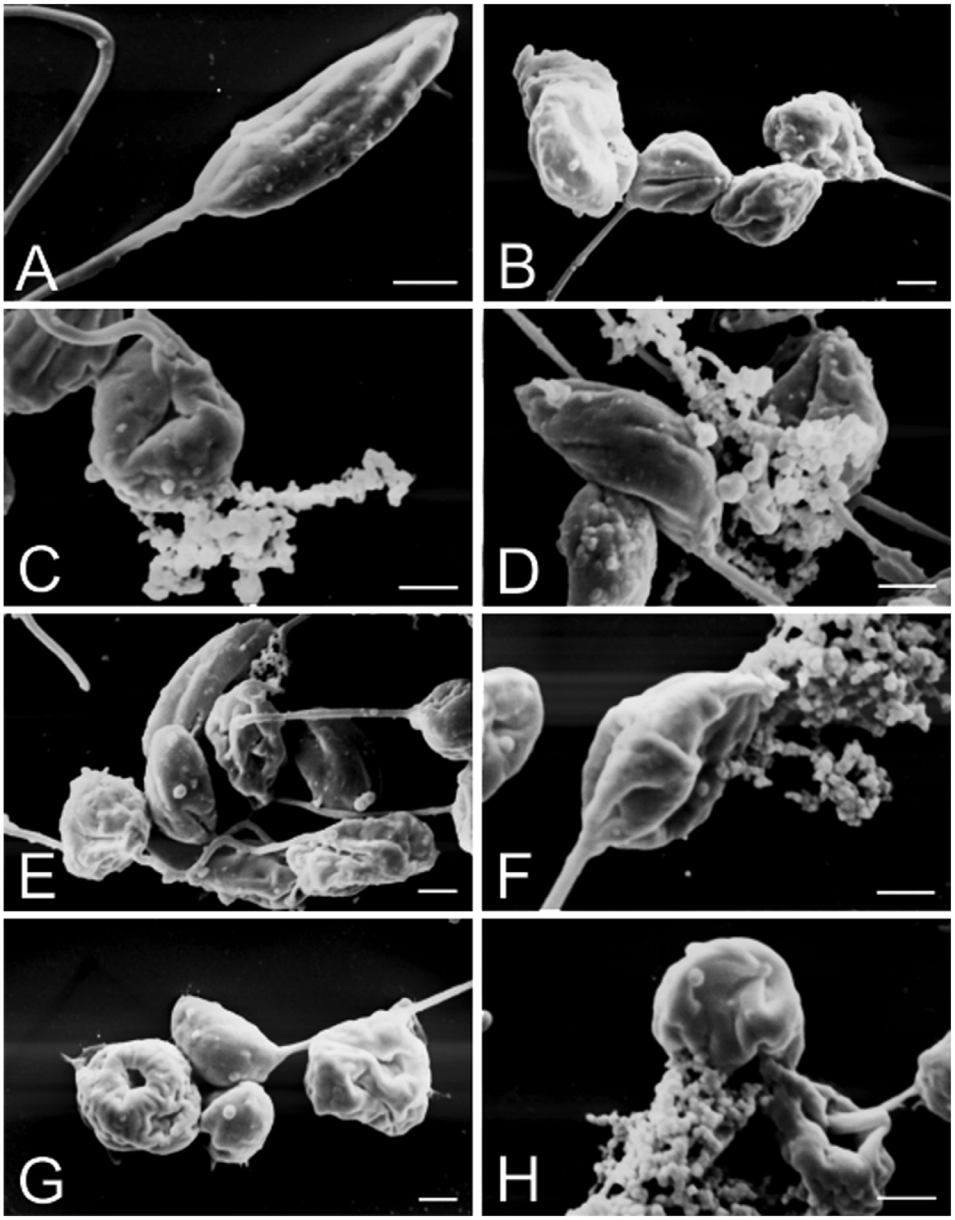
Effect of BenzCo on the morphology of promastigotes incubated for 72 h, observed by scanning electron microscopy. (A) Control. (B-D). Parasites treated with 3.8 µM of BenzCo. (E–H) Parasites treated with 7.8 µM of BenzCo. Bars = 1 µm.

**Figure 5:**
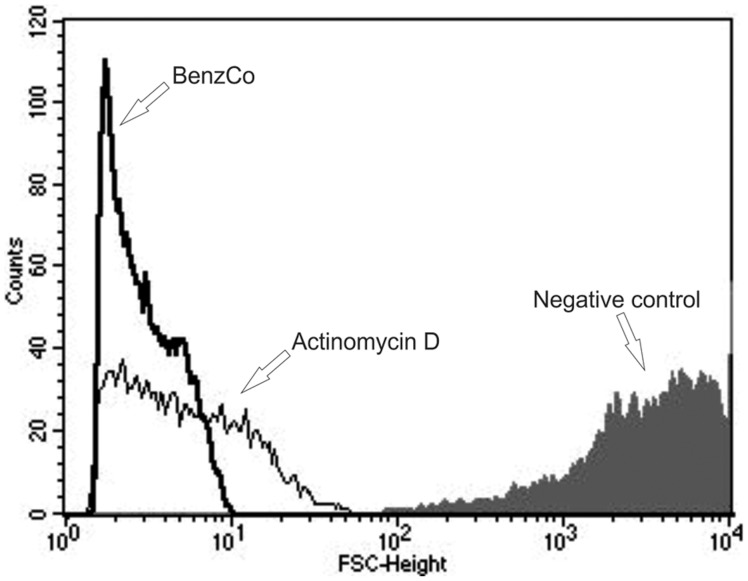
Flow cytometry analysis of promastigote forms of *Leishmania amazonensis* treated with BenzCo for 72 h. Forward light scatter (FSC-H) was considered as function of cell size. Representative FACS histogram showing FSC-H of promastigotes treated with IC_50_ and the control group (untreated cells, gray full histogram). Actinomycin D (20.0 mM) was used as a positive control. Typical histogram of at least three independent experiments.

### 5. In vitro Antiproliferative Activity Assays Against Promastigotes and Axenic Amastigotes

Promastigotes (1×10^6^ parasites per milliliter) were grown in 24-well culture microplates at 25°C in Warren’s medium that contained 10% FBS and various concentrations of BenzoCo. Axenic amastigote forms (1×10^6^ parasites/ml) were grown in 12-well culture microplates at 32°C in Schneider’s medium, supplemented with 20% FBS in the presence of increasing concentrations of the compound and incubated for 72 h. The treatments were performed at final concentrations of 1.3, 6.6, 13.0, 66.0, and 131.0 µM. Amphotericin B was used as a positive control. Dimethyl sulfoxide (DMSO) was used to solubilize the stock solution of the compound. The final DMSO concentration did not exceed 1.0%, which has no deleterious effects on the parasites. Leishmanicidal activity was determined by direct counting of the free-living parasites in Neubauer chamber, and the 50% inhibition concentration (IC_50_) was evaluated graphically by plotting the concentration *vs*. percentage growth inhibition.

**Figure 6:**
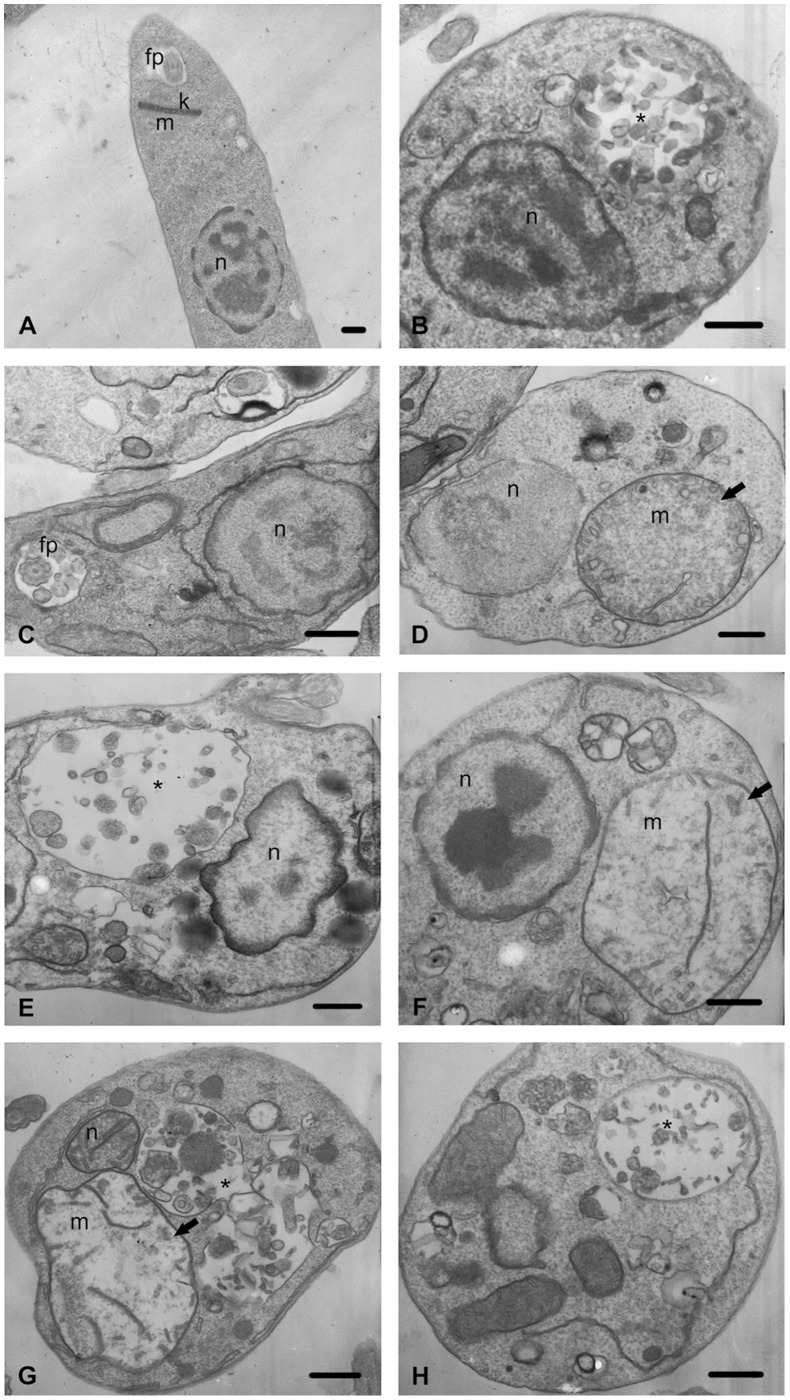
Ultrastructural effect of BenzCo on promastigote forms of *Leishmania amazonensis* after treatment with 3.8 and 7.8 µM of BenzCo for 72 h at 25°C, observed by transmission electron microscopy. (A) Control. (B–E) Parasites treated with 3.8 µM. (E–H) Parasites treated with 7.8 µM. Arrows indicate swollen mitochondria, and the asterisk indicates autophagic vacuoles. f, flagellum; fp, flagellar pocket; k, kinetoplast; m, mitochondrion; n, nucleus. Bars = 1 µm.

### 6. Activity Against Intracellular Amastigotes

Sterile glass coverslips were placed in the wells of a 24-well microplate. Peritoneal macrophages were collected from BALB/c mice by washing with cold PBS. After 5×10^5^ cells per milliliter were plated on cover slips in RPMI 1640 medium supplemented with 10% FBS and incubated for 24 h at 37°C. Promastigotes were then added to the cell monolayer at a 10∶1 parasites:macrophage ratio. After 6 h of interaction, the monolayer cells were washed with RPMI 1640 medium to remove the non-interiorized parasites. After, the infected macrophages were treated with BenzCo at concentrations of 6.6, 13.0, 26.0, and 40.0 µM and incubated for 20 h. The monolayers were then fixed with methanol and stained with 10% Giemsa stain. The percentage of infected macrophages was determined by counting at least 200 macrophages. The results are expressed as the number of parasites/100 macrophages.

**Figure 7:**
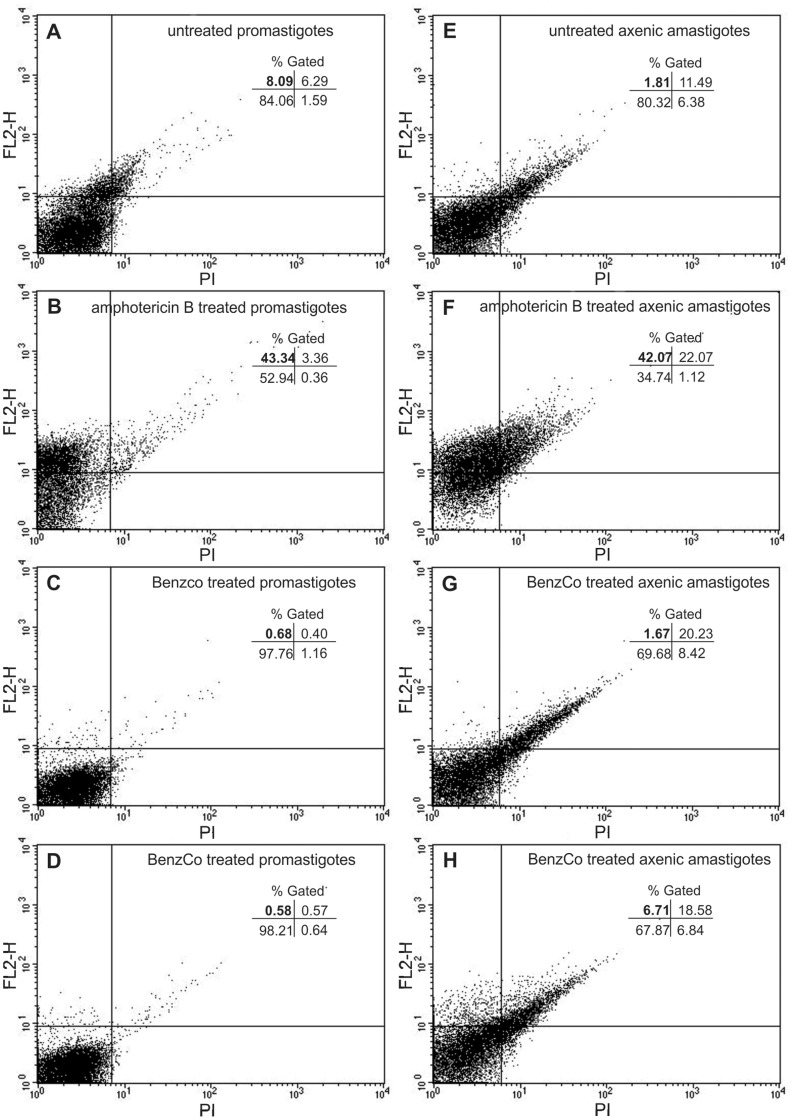
Flow cytometry analysis of *L. amazonensis* treated with BenzCo and stained with propidium iodide (PI). (A) Untreated promastigotes. (B) Promastigotes treated with 5.0 µM amphotericin B. (C and D) Promastigotes treated with 66.0 and 131.0 µM BenzCo, respectively. (E) Untreated axenic amastigotes. (F) Axenic amastigotes treated with 10.0 µM amphotericin B. (G and H) Axenic amastigotes treated with 66.0 and 131.0 µM BenzCo, respectively. The bold numbers show the percentage of PI-positive cells in the upper left quadrant.

### 7. Ultrastructural Analysis

Promastigotes treated with 3.8 and 7.8 µM of BenzCo for 72 h at 25°C were fixed in 2.5% glutaraldehyde in 0.1 M sodium cacodylate buffer for 1–3 h. Subsequently, the cells were adhered on poly-L-lysine-coated coverslips and dehydrated in increasing concentrations of ethanol. The samples were critical-point dried in CO_2_, coated with gold, and observed in a Shimadzu SS-550 scanning electron microscope.

For analysis by transmission electron microscope promastigote forms fixed, as described above, were post-fixed in a solution that contained 1% osmium tetroxide, 0.8% potassium ferrocyanide, and 5 mM calcium chloride, dehydrated in a graded acetone series, and embedded in Epon resin for 72 h at 60°C. Ultrathin sections were stained with 5% uranyl acetate and lead citrate and examined in a Zeiss 900 transmission electron microscope.

**Figure 8:**
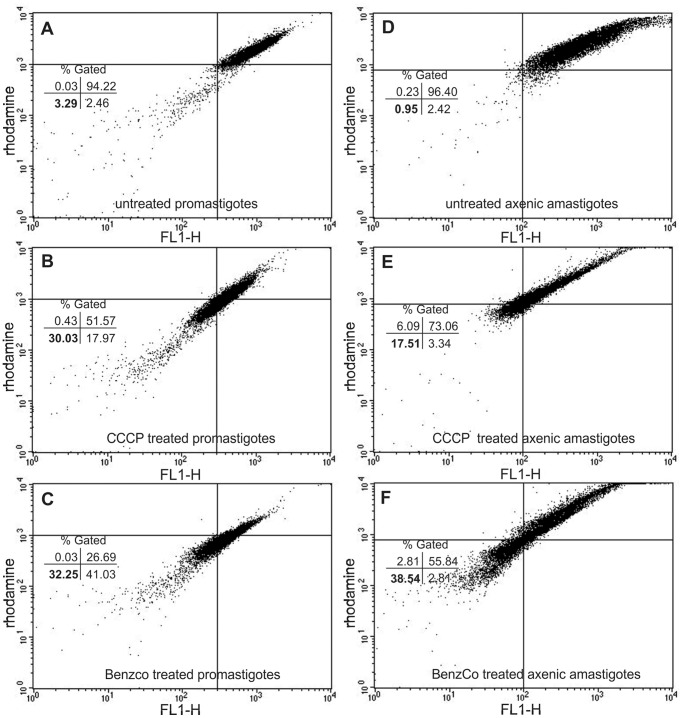
Flow cytometry analysis of Rhodamine 123-labeled (A–C) promastigotes and (D–F) axenic amastigotes of *L. amazonensis.* (A) Untreated promastigotes. (B) Promastigotes treated with 50.0 µM CCCP. (C) Promastigotes treated with 66.0 µM BenzCo. (D) Untreated axenic amastigotes. (E) Axenic amastigotes treated with 200.0 µM CCCP. (F) Axenic amastigotes treated with 197.0 µM BenzCo. The numbers in bold represent the percentage of collapsed ΔΨm cells in the upper right quadrant.

### 8. Determination of Cellular Membrane Integrity

Promastigotes and axenic amastigotes treated or untreated with 66.0 and 131.0 µM BenzCo for 3 h at 32°C were harvested and washed with phosphate-buffered saline (PBS) buffer. The cells were incubated with 50 µl of 2 mg/ml propidium iodide (PI) for 5 min according to the instructions provided by the manufacturer. Immediately thereafter, the cells were analyzed by means of a BD FACSCalibur flow cytometer equipped with Cell Quest software. A total of 10,000 events were acquired in the region that corresponded to the parasites. Amphotericin B at 5.0 and 10.0 µM was used as a positive control.

**Figure 9:**
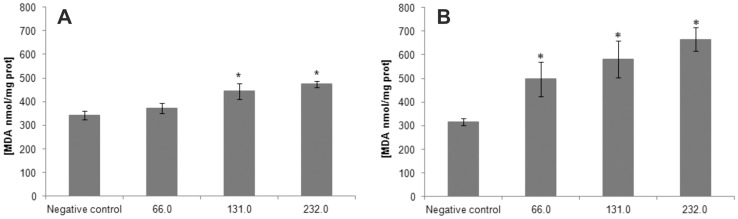
Effect of BenzCo on lipid peroxidation in the (A) promastigote and (B) axenic amastigote forms. Each bar represents the mean ± standard error of at least three independent experiments. *p<0.05, significant difference of each group from control.

### 9. Determination of Mitochondrial Transmembrane Potential (ΔΨm)

Promastigotes and axenic amastigotes treated or untreated with 66.0 and 197.0 µM BenzCo, respectively, for 3 h at 37°C were harvested and washed with PBS. The cells were incubated with 1 µl (5 mg/ml in ethanol) of Rhodamine 123 (Rh 123; Sigma-Aldrich, St. Louis, MO, USA) for 15 min, resuspended in 0.5 ml PBS, and incubated for an additional 30 min. The assay was conducted according to the manufacturer’s instructions. The parasites were analyzed by means of a BD FACSCalibur flow cytometer and Cell-Quest Pro software. A total of 10,000 events were acquired in the region that corresponded to the parasites. Carbonyl cyanide 3-chlorophenylhydrazone (CCCP) at 50.0 and 200.0 µM was used as a positive control.

**Figure 10:**
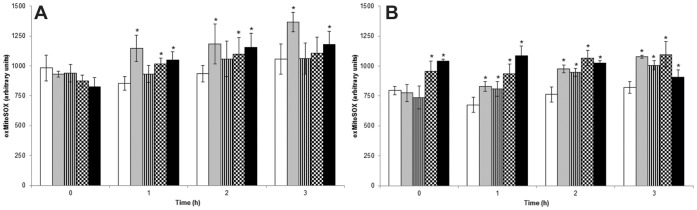
MitoSOX oxidation. The (A) promastigote forms and (B) axenic amastigote forms were incubated with MitoSOX reagent after the parasites were washed three times with KH buffer and untreated (white box, negative control) or treated with BenzCo (striped box, 66.0; squared box, 131.0; and black box, 232.0 µM). Antimycin A (10 µM, light gray box) was used as a positive control. Fluorescence was measured with a VICTOR X3^TM^spectrofluorometer (Perkin-Elmer). The results are expressed as the mean ± standard error of arbitrary units of MitoSOX oxidation from three independent experiments.

### 10. Measurement of Lipid Peroxidation Product

Promastigotes and axenic amastigotes in exponential phase were treated with 66.0, 131.0, and 232.0 µM BenzCo for 6 h. After treatment, the cells were washed with phosphate buffer, homogenized, and added to a solution of 0.37% thiobarbituric acid in 15% trichloroacetic acid and 0.25 N HCl. The mixture was heated at 90–95°C for 45 min. After cooling, butanol (1∶1) was added to the solution. The mixture was shaken and centrifuged at 2,000×*g* for 5 min. The optical density of the organic layer was determined at 535 nm in a BIO-TEK Power Wave XS spectrophotometer. Lipid peroxidation was determined by the generation of thiobarbituric acid-reactive substances (TBARS) in terms of malondialdehyde (MDA), expressed as nanomoles of MDA per milligram of protein [16]. Each experiment was conducted in duplicate and repeated at least three times.

### 11. Determination of Cell Volume of Parasites

Promastigotes treated with the IC_50_ concentration of BenzCo for 72 h at 25°C were harvested and washed with PBS. Subsequently, the parasites were analyzed by means of a BD FACSCalibur flow cytometer and Cell-Quest Pro software. Histograms and analysis were performed, FSC-H which represents the cell volume. A total of 10,000 events were acquired in the region that corresponded to the parasites. Actinomycin D at 20.0 mM was used as a positive control.

### 12. Fluorimetric Detection of Mitochondrial Superoxide Anion Radical (O_2_^•−^ )

Promastigotes and axenic amastigotes were harvested and washed with Krebs-Henseleit (KH) solution buffer, pH 7.3 that contained 15 mM NaHCO_3_, 5 mM KCl, 120 mM NaCl, and 0.7 and 1.5 mM NaH_2_PO_4_. The cells were loaded with 2.0 ml of 5 µM MitoSOX reagent [3,8-phenanthridinediamine, 5-(6-triphenylphosphoniumhexyl)-5,6-dihydro-6-phenyl]. The parasites were incubated for 10 min at 37°C and protected from light. After incubation with MitoSOX reagent, the parasites were washed three times with KH buffer and treated or untreated with 66.0, 131.0, and 232.0 µM BenzCo. Antimycin A at 10 µM was used as a positive control. MitoSOX detection was performed using black 96-well plates for 3 h. Fluorescence was measured at excitation and emission wavelengths of 510 and 580 nm, respectively, in a VICTOR X3™ spectrofluorometer (Perkin Elmer). The results are expressed as arbitrary units of MitoSOX [17].

### 13. Statistical Analysis

In the cellular experiments, the 50% growth inhibition value (IC_50_) was determined from the linear concentration-response curves, and the results are expressed as the mean and standard deviation of at least three independent experiments. Parametric data were analyzed using one-way analysis of variance (ANOVA), and significant intergroup differences were analyzed using Dunnett’s test. Nonparametric data were analyzed using the Kruskal-Wallis test. All statistical analyses were performed at the *p*<0.05 level of significance.

## Results

### 1. Antileishmanial Activity

The treatment of the parasites with the synthesized metallic compound resulted in the dose-dependent growth inhibition of the promastigote and axenic amastigote forms of *L. amazonensis*. After direct counting of the free-living parasites in Neubauer chamber, was calculated the inhibition percentage of the parasites, and the concentration corresponding to 50% and 90% inhibition of the parasites were obtained by plotting the concentration *vs*. percentage growth inhibition for linear regression. At concentrations above 13.0 µM, the compound completely inhibited the growth of promastigotes. At concentrations above 66.0 µM, the compound inhibited the growth of axenic amastigote forms (Fig. 2). The IC_50_ concentrations of BenzCo in promastigotes and axenic amastigotes after 72 h of incubation were 3.8 µM and 9.5 µM, respectively. Amphotericin B had IC_50_ values of 0.063 µM and 0.249 µM against the promastigote and axenic amastigote forms, respectively. Copper salt (CuCl_2_) was also assessed against promastigote forms and displayed low activity compared with its complex, with an IC_50_ value of 302.1 µM, indicating that copper alone inhibited parasite growth only at concentration about 10 times higher, but the complex was important for obtaining an active organometallic compound.

### 2. Effect on Intracellular Amastigotes

The effects of BenzCo on intracellular amastigotes were observed after 24 h of incubation (Fig. 3). BenzCo exerted activity at an IC_50_ value of 10.7 µM. The numbers of parasites/100 macrophages were 136.8 at 6.6 µM, 98.9 at 13.0 µM, 57.7 at 26.0 µM, and 31.6 at 40.0 µM. These results correspond to survival percentages of 62.3%, 45.0%, 26.3%, and 14.4%, respectively.

### 3. Scanning Electron Microscopy

Morphological alterations in promastigote forms treated with BenzCo were observed by scanning electron microscopy. Photomicrographs revealed that untreated protozoa showed typical characteristics, with an elongated shape and terminal flagellum. BenzCo dose-dependently altered the shape and size of the treated parasites, including cellular disintegration (Fig. 4). To further confirm the alterations in cell shape and size in promastigote forms shown by SEM, the cell was assessed by flow cytometry. The histogram revealed that parasites treated with BenzCo showed a reduction in the size of the parasites (Fig. 5).

### 4. Transmission Electron Microscopy

To investigate the effects of BenzCo on ultrastructure, promastigotes were incubated for 72 h in the presence of BenzCo and then analyzed by transmission electron microscopy (TEM). BenzCo induced different alterations in the ultrastructure of promastigotes, sometimes producing dramatic changes in the mitochondrial structure, changes in the appearance of typical autophagic structures and vacuolization of the parasite’s cytoplasm (Fig. 6).

### 5. Cellular Membrane Integrity

Plasma membrane integrity in promastigotes and axenic amastigotes was determined by staining with propidium iodide (PI), which diffuses across permeable membranes and binds to nucleic acids. Following treatment of promastigotes with BenzCo at 66.0 µM and 131.0 µM, the gated percentage of PI-stained parasites decreased to 0.68% and 0.58%, respectively (Fig. 7C and D, upper-left quadrant). Untreated promastigotes showed the percentage of gate cells at 8.09% (Fig. 7A, upper-left quandrant). In contrast, promastigotes treated with 5 µM amphotericin B showed a increased in the gated percentage of PI-stained cells (43.34%, upper left quadrant, Fig. 7B). Treated axenic amastigotes showed PI binding of 1.67% and 6.71% (upper left quadrant) at 66.0 and 131.0 µM BenzCo, respectively (Fig. 7G and H). These results were similar to the negative control (1.81%, untreated cells, Fig. 7E). In contrast, axenic amastigotes treated with 10 µM amphotericin B (positive control), showing an increase in the gated percentage of PI-stained cells of 42.07% (Fig. 7F, upper left quadrant). This indicates that labeling BenzCo-treated promastigotes and axenic amastigotes with PI did not show permeabilization of the plasma membrane.

### 6. Membrane Mitochondrial Potential (ΔΨm)

The TEM ultrastructural analysis demonstrated that BenzCo induced alterations in the mitochondria of treated promastigotes, and we decided to evaluate the mitochondrial membrane potential by flow cytometry using Rh 123, a fluorescent marker that indicates mitochondrial membrane potential. When promastigotes treated with 66.0 µM BenzCo were labeled with Rh 123, was observed a marked decrease in the percentage population of upper right quadrant gate (26.69%, Fig. 8C). This indicates depolarization of the mitochondrial membrane potential. Similarly, promastigotes treated with CCCP showed a decrease in membrane potentials (51.57%, Fig. 8B). In contrast, untreated parasites maintained the membrane potential (94.22%, upper right quandrant, Fig. 8A). Additionally, axenic amastigotes treated with 197.0 µM and with 200 µM CCCP also showed a decrease in the percentage population of upper right quadrant gate (55.84% and 73.06 respectively, Fig. 8F–E), and untreated axenic amastigotes maintained the membrane potential (96.40%, Fig. 8D).

### 7. Lipid Peroxidation

Lipid peroxidation was assessed by measuring TBARS in promastigotes and axenic amastigotes after treatment with different concentrations of BenzCo compared with control and untreated cells (Fig. 9). The TBARS measurement revealed a dose-dependent effect of BenzCo on promastigotes. Treatment with 66.0, 131.0, and 232.0 µM induced 1.1-, 1.3-, and 1.4-fold increases in lipoperoxidation compared with controls. The same treatment in axenic amastigotes resulted in 1.6-, 2.2-, and 2.2-fold increases in lipoperoxidation, respectively.

### 8. Mitochondrial O_2_^• -^ Production

Reactive oxygen species (ROS) production was evaluated using MitoSOx reagent that measures the mitochondrial accumulation of superoxide, reflecting ROS levels in mitochondria. MitoSOX localized to the mitochondrion because of its hydrophobic nature and its positively charged triphenylphosphonium moiety (Fig. 10). MitoSOX oxidation was higher in BenzCo-treated parasites compared with controls. In promastigotes, the increase in MitoSOX oxidation was observed after 1 h of incubation, and the treatment with 131.0 and 232.0 µM showed more MitoSOX oxidation. In axenic amastigotes, all of the concentrations tested (66.0, 131.0, and 232.0 µM) showed MitoSOX oxidation after 1 h of incubation.

## Discussion

Leishmaniasis causes high levels of morbidity and mortality, principally in the tropics and subtropics. *Leishmania* is an intracellular parasite in the mammalian host and is involved in pathologies that range from cutaneous to visceral forms, depending on the species and the host’s immune response [2], [18]. Extensive studies of new drugs with antileishmanial activity, including both natural products and synthetic compounds, have been performed worldwide [19], [20]–[24].

Researchers have conducted *in vitro* and *in vivo* assays with the aim of finding new active compounds. Studies on the morphological and metabolic pathways in this protozoan have contributed to the elucidation of targets for drug action [25], [26]. In the present study, we evaluated the antileishmanial activity of benzaldehyde thiosemicarbazone derived from limonene complexed with copper against *L. amazonensis*. This compound inhibited the growth of the promastigote, axenic amastigote, and intracellular amastigote forms of the parasite. Thiosemicarbazones constitute an important class of synthetic compounds with several pharmacological activities, which have been the subject of intensive study [5].

Mitochondria in trypanosomatid parasites are attractive chemotherapeutic targets because they have structural and functional characteristics that are distinct from mammalian cells [27]. Mitochondria are an important cellular source for the generation of ROS inside the cells. The maintenance of mitochondrial transmembrane potential (ΔΨm) is essential for the survival of the cell because it derives the synthesis of adenosine triphosphate and maintains oxidative phosphorylation [28]. The reduction of oxygen that occurs at various sites along the mitochondrial respiratory electron chain generates free radicals, such as superoxide radicals [29].

In the present study, BenzCo produced ultrastructural alterations in L. amazonensis promastigotes, mainly on mitochondria, such as swollen mitochondria as well as a autophagic process and vacuolization of the cytoplasm. However, the cytoplasmic membrane apparently remained unaltered.

Mitochondrial damage, with concentric membranes within this organelle and in the flagellar pocket, and the formation of autophagic structures may be associated with ergosterol depletion and alterations in the physical properties of the membranes [30]. The presence of protrusions in the flagellar pocket, apparently formed by the plasma membrane and then released from the cell body with a portion of the cytoplasm, indicates a change in the plasticity of the membrane that lines this flagellar pocket from its normal arrangement, allowing the formation of exocytic projections [31].

In the present study, no alterations in the plasma membrane were detected by flow cytometry using PI as a marker. In contrast, promastigotes and axenic amastigotes treated with BenzCo and stained with Rh 123 showed a decrease in membrane potentials compared with untreated parasites, indicating mitochondrial membrane depolarization. Depolarization of mitochondrial membrane potential in the cells suggests interference with the hydrogen-ion potential of the mitochondrial membrane, similar to reports of *L. amazonensis* treated with lysophospholipid analogs [30]. Additionally, a decrease in Rh 123 fluorescence could be related to interference with the proton electrochemical potential gradient of the mitochondrial membrane [32]. A previous study demonstrated that quercetin exerts its antileishmanial effect on *L. amazonensis* promastigotes by generating ROS and affecting parasite mitochondrial function [33].

Alternatively, damage to the lipid bilayer of mitochondria caused by ROS could eventually lead to a leak of cytochrome c, which is one of the key events that lead to apoptosis [34]. The percentage of PI fluorescence in parasites labeled after treatment with BenzCo in the present study was similar to the percentage in untreated parasites. Together with the results observed with mitochondrial membrane potential, these results are consistent with the extensive damage to the parasite mitochondrion detected by TEM. Additionally, the treatment of the parasites with BenzCo dose-dependently formed ROS and increased lipoperoxidation, and axenic amastigotes were more susceptible than promastigotes to lipoperoxidation and mitochondrial O_2_^•−^ production. Previous studies have demonstrated that mitochondrial membrane potential induces the formation of ROS inside cells and lipid peroxidation [35]–[37]. In conclusion, the *in vitro* antileishmanial activity of BenzCo was observed, and this activity may be associated with mitochondrial dysfunction and increased ROS generation, leading to parasite death.
